# A novel motif in the proximal C-terminus of Pannexin 1 regulates cell surface localization

**DOI:** 10.1038/s41598-019-46144-5

**Published:** 2019-07-05

**Authors:** Anna L. Epp, Sarah N. Ebert, Juan C. Sanchez-Arias, Leigh E. Wicki-Stordeur, Andrew K. J. Boyce, Leigh Anne Swayne

**Affiliations:** Division of Medical Sciences and Island Medical Program, University of Victoria, Victoria, V8P 5C2 Canada

**Keywords:** Membrane trafficking, Ion channels

## Abstract

The Pannexin 1 (Panx1) ion and metabolite channel is expressed in a wide variety of cells where it regulates a number of cell behaviours including proliferation and differentiation. Panx1 is expressed on the cell surface as well as intracellular membranes. Previous work suggests that a region within the proximal Panx1 C-terminus (Panx1CT) regulates cell surface localization. Here we report the discovery of a putative leucine-rich repeat (LRR) motif in the proximal Panx1CT necessary for Panx1 cell surface expression in HEK293T cells. Deletion of the putative LRR motif results in significant loss of Panx1 cell surface distribution. Outcomes of complementary cell surface oligomerization and glycosylation state analyses were consistent with reduced cell surface expression of Panx1 LRR deletion mutants. Of note, the oligomerization analysis revealed the presence of putative dimers and trimers of Panx1 at the cell surface. Expression of Panx1 increased HEK293T cell growth and reduced doubling time, while expression of a Panx1 LRR deletion mutant (highly conserved segment) did not reproduce this effect. In summary, here we discovered the presence of a putative LRR motif in the Panx1CT that impacts on Panx1 cell surface localization. Overall these findings provide new insights into the molecular mechanisms underlying C-terminal regulation of Panx1 trafficking and raise potential new lines of investigation with respect to Panx1 oligomerization and glycosylation.

## Introduction

Pannexin 1 (Panx1) is a four-transmembrane domain protein that oligomerizes in hexamers to form ion and metabolite channels, and is detected at the cell surface and intracellular membranes (reviewed in Boyce *et al*.^1^). The factors regulating Panx1 trafficking and cell surface localization have been the focus of intense study, identifying glycosylation state^2–4^ and the intracellular C-terminus^5–7^ as important contributors. The cumulative data of several studies suggests that the Panx1 C-terminus (Panx1CT) is required, but not sufficient for cell surface localization. Panx1CT deletion in BICR-M1R_k_ cells resulted in a loss of Panx1 cell surface localization^6^. Furthermore, substitution of the Panx2 C-terminus with that of Panx1 was not sufficient to direct the usually intracellular Panx2 to the cell surface in Neuro-2a (N2a) cells^7^. In addition, distal Panx1CT deletion mutants retain cell surface expression and/or function that is dependent on surface localization^8,9^. Together these previous studies suggest a region in the proximal C-terminus is important for trafficking of Panx1 to the cell surface.

In the present study, we seek to expand our understanding of Panx1CT regulation of cell surface expression. We created EGFP-tagged Panx1CT deletion mutants and examined their surface expression along with two other key properties that provide additional insight: glycosylation and oligomerization state. Panx1 exhibits 3 main glycosylation states: Gly0 (unglycosylated), Gly1 (high-mannose species), and Gly2 (complex glycosylation)^2–4^. The complex glycosylation species of wildtype Panx1 is more frequently associated with cell surface expression^2–4^, providing an important additional molecular signature when assessing surface expression. In terms of oligomerization state, it is not yet known how Panx1 cell surface expression and hexamerization^3,10,11^ are linked; one possibility is that Panx1 reaches the cell surface prior to oligomerization, which could have important implications for its functional properties^10^. We therefore investigated the localization, glycosylation, and oligomerization profiles of EGFP-tagged Panx1CT deletion mutants in HEK293T cells, which lack appreciable endogenous Panx1 expression at the protein level (however, see Sanchez-Pupo *et al*.^12^).

Our new data suggest that the proximal Panx1CT (residues R299-D378) is necessary for Panx1 cell surface localization. Within this region we identify the consensus sequence of a novel 21 amino-acid long putative leucine-rich repeat (LRR; S328-K348) motif. In general, multiple LRR motifs combine to form LRR domains, which are recognized for their ability to mediate interaction with other proteins or lipids (reviewed in Bella *et al*.^13^), to influence protein localization to specific subcellular compartments, and facilitate cell-cell contacts, amongst other roles. For example, in LRR and PDZ (LAP; PDZ, PSD-95/Discs-large-ZO1) proteins, the LRR domain is required for targeting to the basolateral membrane of epithelial cells^14^. We thus investigated the role of the putative C-terminal LRR motif in Panx1 cell surface localization using several complementary techniques (cell surface biotinylation, confocal microscopy, deglycosylation, cell surface oligomerization and cell proliferation analyses). Overall, this study provides important new insights into the role of the Panx1CT in cell surface localization, identifying a novel, putative LRR motif within the proximal C-terminus that is required for cell surface localization.

## Results

### A region in the proximal Panx1CT is required for cell surface localization

**Figure 1:**
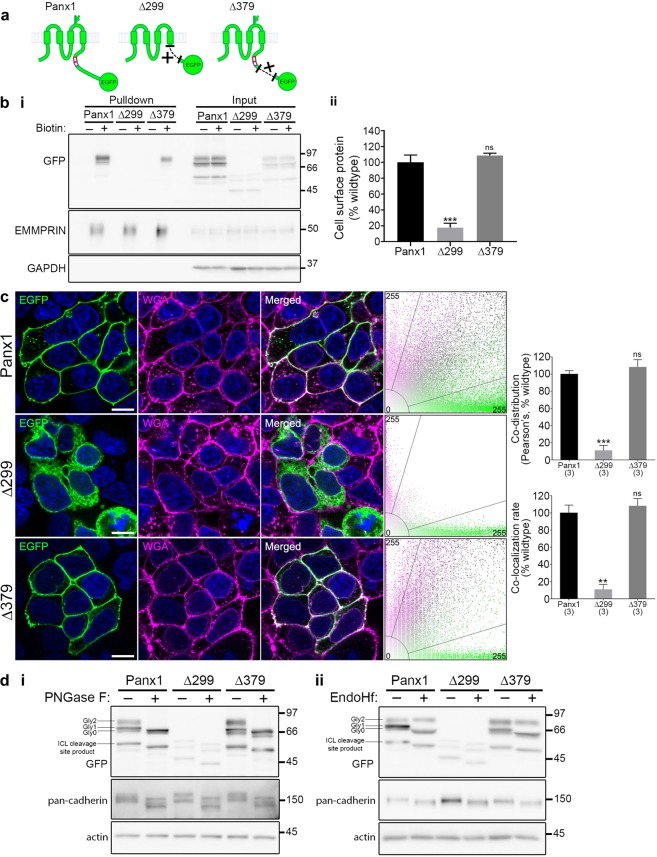
The proximal Panx1CT is necessary for cell surface localization of Panx1-EGFP. (**a**) Schematic of full length Panx1-EGFP and the Panx1∆299-EGFP and Panx1∆379-EGFP deletion mutants. (**b**) Cell surface biotinylation assays reveal that the proximal Panx1CT is required for cell surface localization in HEK293T cells. (*i*) Representative Western blot of pulldown (cell surface protein) and input. Anti-EMMPRIN was used as a positive control for biotin pulldown and as a loading control, and anti-GAPDH was used as a negative control against biotin internalization. (*ii*) Panx1∆299-EGFP exhibited reduced cell surface levels compared to Panx1-EGFP, while surface levels of Panx1∆379-EGFP were similar to those of Panx1-EGFP. Data are presented as mean ± SEM. One-way ANOVA with Dunnett’s multiple comparisons test*, N* = 3, α = 0.05, *F*(2,6) = 62.39, **P = 0.0002, ns, non-significant. (**c**) Representative confocal micrographs of HEK293T cells overexpressing Panx1-EGFP, Panx1∆379-EGFP, or Panx1∆299-EGFP (green). Hoechst was used as a nuclear counterstain (blue) and wheat-germ agglutinin (WGA) was used as a plasma membrane marker (magenta). Overlapping EGFP and WGA signals (white) and scatterplots with EGFP and WGA fluorescence signals show the co-distriution of these proteins along the cell membrane. Panx1-EGFP and Panx1∆379-EGFP co-distributed with WGA, while Panx1∆299-EGFP did not. One-way ANOVA with Dunnett’s multiple comparison test, *N* = 3, α = 0.05; Pearson’s: *F*(2,6) = 62.29, ***P = 0.0002, ns, non-significant; Colocalization rate: *F*(2,6) = 26.81, **P = 0.0014, ns, non-significant. Scale bars, 10 μm. Data are presented as mean ± SEM. (d) Deglycosylation assays using (*i*) PNGase F or (*ii*) EndoHf reveal Panx1-EGFP and Panx1∆379-EGFP exhibited Gly0, Gly1, and Gly2 glycosylation species, while Panx1∆299-EGFP exhibited only Gly0 and Gly1 forms. Anti-pan-cadherin and anti-β-actin were used as a positive and negative controls for deglycosylation, respectively. This figure was modified from Epp 2019^46^. For uncropped images of all Western blots in this figure, please see Supplementary Fig. S3.

We first generated constructs with large deletions of the C-terminus (Fig. 1a): the entire C-terminus (Panx1∆299-EGFP) and the region distal to a C-terminal caspase cleavage site (Panx1∆379-EGFP; D378 is the terminal Panx1 amino acid in this deletion mutant). We used cell surface biotinylation and confocal imaging to determine the localization of Panx1-EGFP and these Panx1CT deletion mutants. There was no significant difference between surface levels of Panx1-EGFP and Panx1∆379-EGFP, consistent with previous reports^8^ and Panx1∆379-EGFP-expressing cells appeared to be perfectly healthy. However, we detected substantially less Panx1∆299-EGFP at the cell surface (Fig. 1b). Similarly, confocal imaging revealed co-distribution of Panx1-EGFP and Panx1∆379-EGFP with the surface marker wheat-germ agglutinin (WGA) while Panx1∆299-EGFP did not co-distribute with WGA as revealed by Pearson’s correlation coefficient analysis, a measure of the strength of the association between the fluorescence intensities of EGFP and WGA (Fig. 1c). We also investigated the glycosylation state of these C-terminus deletion mutations using deglycosylation enzymes that target immature (high-mannose) and mature (complex) glycosylation states. The glycosylation states of Panx1-EGFP and Panx1∆379-EGFP were similar to one another (Fig. 1d). The Gly1 and Gly2 bands in Panx1-EGFP and Panx1∆379-EGFP samples were sensitive to PNGase F treatment (Fig. 1di), confirming complex N-linked glycosylation, which has been previously associated with cell surface expression^2–4^. However, only Gly0 and Gly1 forms were detected for Panx1∆299-EGFP (Fig. 1d), which exhibited reduced expression levels (Supplementary Fig. S1), as previously reported^6^. Together these data confirm that the proximal C-terminus is required for cell surface localization of Panx1, as suggested by previous reports^6,8,9^.

**Figure 2:**
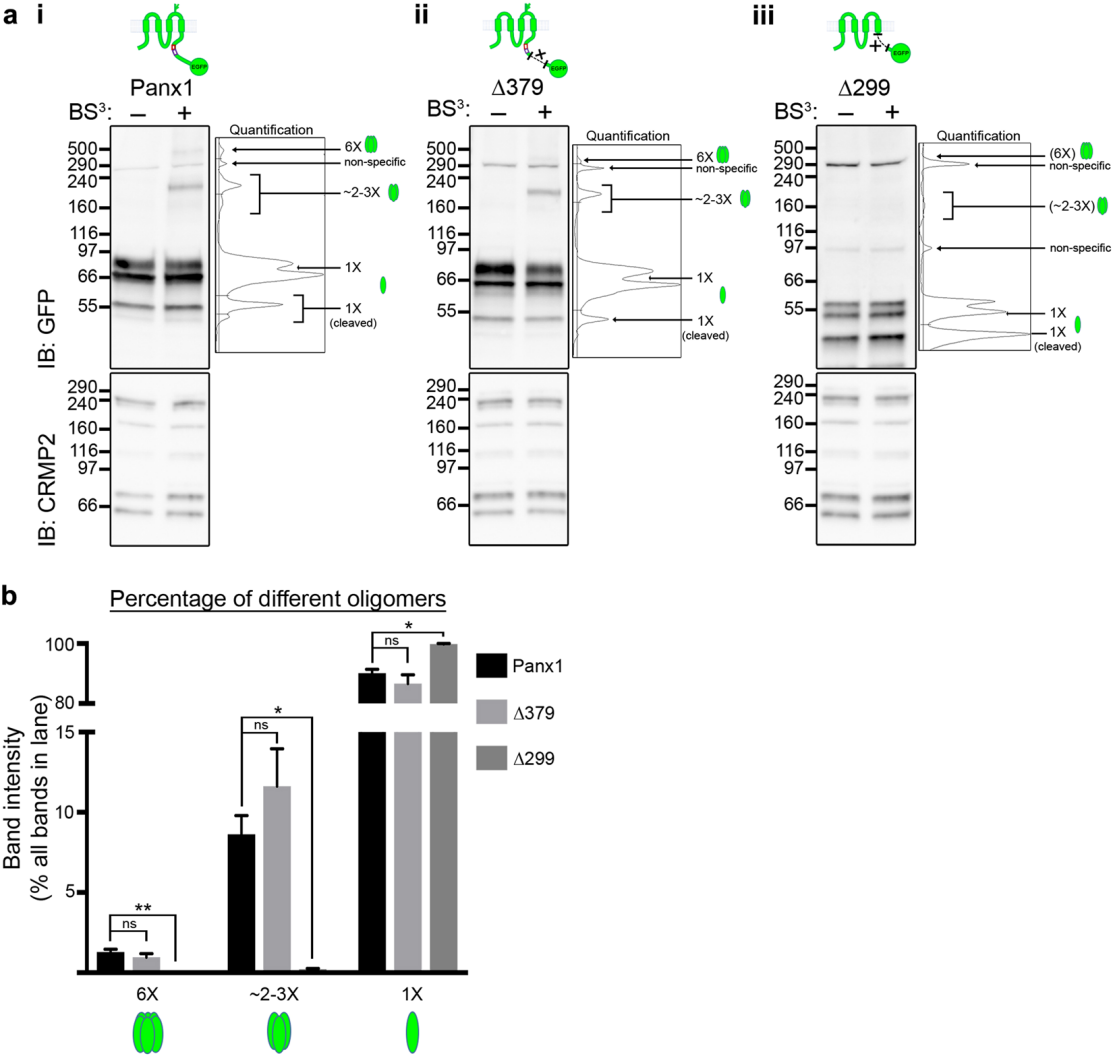
Surface oligomerization is preserved with deletion of the distal Panx1CT. (**a**) Crosslinking assays reveal oligomerization profiles of C-terminal deletion mutants. Representative Western blots observing HEK293T cell lysates post-transfection with (*i*) Panx1-EGFP, (*ii*) Panx1∆379-EGFP, or (*iii*) Panx1∆299-EGFP, and after crosslinking with the cell-impermeable crosslinker, BS^3^, to observe surface-localized oligomers (6× or ~2–3×). The plot of each quantification is included to shed light on the analytical process. An antibody for Crmp2, an intracellular protein known to form tetramers, was used as a negative control to ensure BS^3^ had not entered the cell. Crmp2 oligomerization did not increase in the presence of BS^3^, as expected. (**b**) Each oligomeric band was quantified and expressed as a percentage of the entire GFP signal in each lane. Non-specific bands were excluded from the analysis. Mutants lacking the distal Panx1CT (Panx1∆379-EGFP) had the same oligomerization profiles as full length Panx1-EGFP. Data are presented as mean ± SEM. One-way ANOVA with Dunnett’s multiple comparisons test*, N* = 3, α = 0.05; *F*(2,6) = 17.59, **P = 0.0023 (6×); *F*(2,6) = 15.73, *P = 0.0127 (3×); *F*(2,6) = 13.84, *P = 0.0169 (1×); ns, non-significant. All samples were derived from the same experiment and processed in parallel. This figure was modified from Epp 2019^46^. For uncropped images of all Western blots in this figure, please see Supplementary Fig. S4

To compare the cell surface oligomerization properties of Panx1∆379-EGFP and Panx1-EGFP, we used a cell-impermeable crosslinker, BS^3^ (Fig. 2). A similar deletion mutant, Panx1∆378^8,9^ formed functional cell surface channels implying it is able to oligomerize ‘appropriately’. Panx1-EGFP formed hexamers as previously reported^3,10,11^, as well as intermediate oligomers (corresponding to dimers or trimers). The Panx1∆379-EGFP mutant formed oligomers that corresponded to those of Panx1-EGFP (relative to molecular weight of the monomer; Fig. 2aii,b). That is, there were no significant differences in relative abundance of hexamers, or intermediate oligomers. It is important to note that under these conditions we were not able to discern whether monomeric bands originated from intracellular or cell surface pools. We did not detect high molecular weight species corresponding to intermediate oligomers or hexamers with Panx1∆299-EGFP, which was expected given its restricted cell surface localization required for BS^3^ exposure (Fig. 2aiii,b). We did not detect any higher molecular weight bands with longer exposures (data not shown). These results confirm that cell surface proteins formed oligomers in similar abundance, i.e. oligomerization profile was conserved with the deletion of the distal C-terminus. Overall, these results suggest that a region within the proximal Panx1CT is required for its trafficking to the cell surface and that the distal Panx1CT is dispensable for oligomerization of Panx1. We thus asked whether there are specific amino acids in the proximal Panx1CT that regulate Panx1 trafficking.

### A putative leucine-rich repeat motif in the proximal Panx1CT is required for cell surface localization

**Figure 3:**
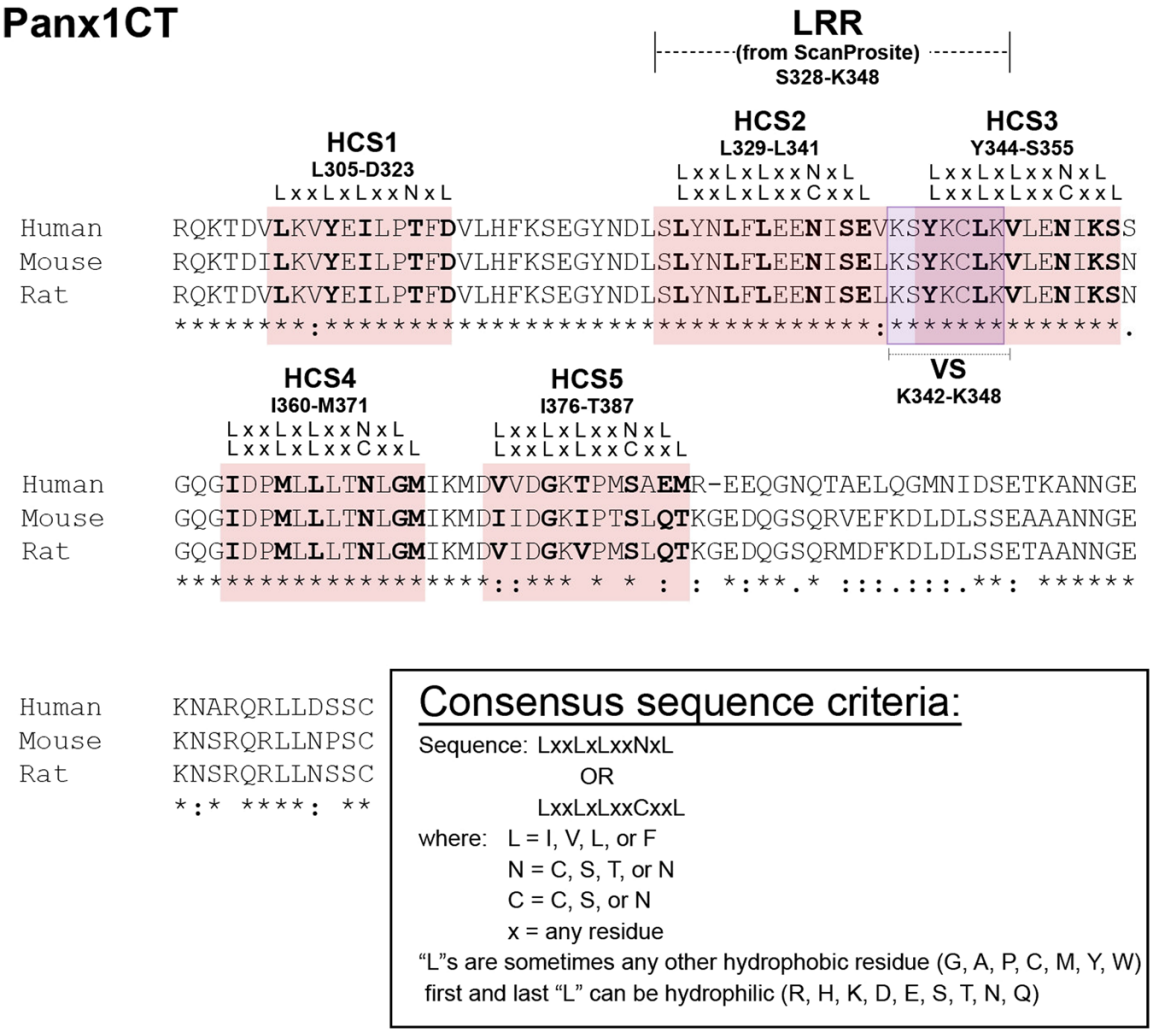
The Panx1CT amino acid sequence contains 5 LRR HCS consensus sequences. Sequence alignment of Panx1CT from human, mouse, and rat. Each of the identified HCS regions are indicated in red, and labeled with the corresponding consensus sequence. The full LRR identified by ScanProsite was separated into an HCS and VS (the VS is indicated in purple). The alignment was generated using CLUSTAL O (1.2.3) with NCBI accession numbers: NP_056183.2 (human), NP_062355.2 (mouse), and NP_955239.1 (rat). This figure was modified from Epp 2019^46^.

The ScanProsite tool^15^ was used to detect previously unidentified motifs within the mouse Panx1 amino acid sequence (NCBI accession number: NP_062355.2) that could contribute to Panx1 localization. The scan (using high-sensitivity settings) uncovered a previously unreported putative leucine-rich repeat (LRR) motif at residues S328-K348 (Fig. 3). Further examination revealed the putative LRR motif perfectly fit the criteria: single LRRs typically consist of a highly conserved segment (HCS) of 11–12 residues consisting of LxxLxLxxNxL or LxxLxLxxCxxL; where L is Leu, Ile, Val, or Phe (but can be replaced by any hydrophobic amino acid); N is Asn, Thr, Ser, or Cys; and C is Cys, Ser, or Asn; and where the first and last L can be replaced by relatively hydrophilic residues, followed by a variable segment (VS)^13,16–20^. As multiple (2 or more) LRR motifs are usually found in relatively close proximity in a protein sequence to form solenoidal LRR domains^13,20^, we further inspected the amino acid sequence for adjacent LRR motifs within the Panx1CT. We discovered an additional 4 sequences consistent with the HCS consensus criteria and numbered each segment HCS1-HCS5 (Fig. 3). These sequences are conserved between mouse, human, and rat Panx1CT. As these additional HCS sequences were not identified by ScanProsite, we were not able to identify prospective VS included within each corresponding LRR motif.

**Figure 4:**
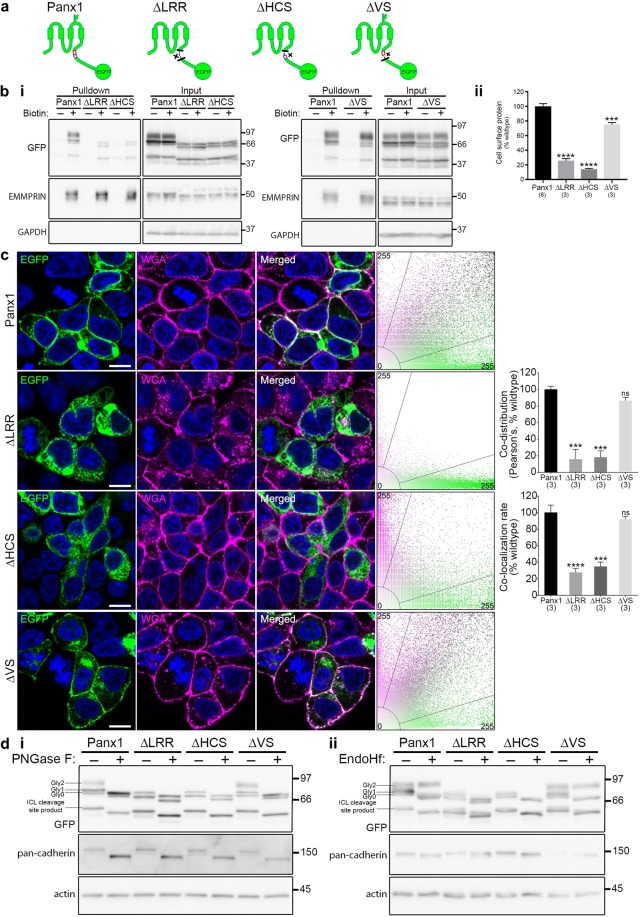
A novel putative LRR motif in the Panx1CT is necessary for trafficking Panx1-EGFP to the cell surface. (**a**) Schematic of full length Panx1-EGFP and the Panx1∆LRR-EGFP, Panx1∆HCS-EGFP, and Panx1∆VS-EGFP deletion mutants. (**b**) Cell surface biotinylation assays reveal that the putative LRR motif, or HCS alone, is required for cell surface localization, while the VS is also mildly important. (*i*) Representative Western blot of pulldown (cell surface protein) and input. Anti-EMMPRIN was used as a positive control for biotin pulldown and as a loading control, and anti-GAPDH was used as a negative control against biotin internalization. (*ii*) Panx1∆LRR-EGFP and Panx1∆HCS-EGFP exhibited markedly reduced cell surface levels compared to Panx1-EGFP, while Panx1∆VS-EGFP exhibited a relatively small reduction cell surface levels compared to Panx1-EGFP. Data are presented as mean ± SEM. One-way ANOVA with Dunnett’s multiple comparisons test*, N* = 3, α = 0.05, *F*(3, 11) = 152.4, ***P = 0.0007, ****P < 0.0001, ns, non-significant. (**c**) Confocal images of HEK293T cells overexpressing Panx1-EGFP, Panx1∆LRR-EGFP, Panx1∆HCS-EGFP, or Panx1∆VS-EGFP (green). Hoechst was used as a nuclear counterstain (blue) and wheat-germ agglutinin (WGA) was used as a plasma membrane marker (magenta). Overlapping EGFP and WGA signals (white) and scatterplots with EGFP and WGA fluorescence signals show the co-distribution of these proteins along the cell membrane. While both Panx1-EGFP and Panx1∆VS-EGFP co-distributed with WGA, Panx1∆LRR-EGFP and Panx1∆HCS-EGFP did not. *N* = 3, α = 0.05; Pearson’s: *F*(2,6) = 32.94, ***P = 0.0002, ns, non-significant; Colocalization rate: *F*(2,6) = 38.99, ***P = 0.0002, ****P < 0.0001, ns, non-significant. Scale bars, 10 μm. Data are presented as mean ± SEM. (d) Deglycosylation assays using (*i*) PNGase F or (*ii*) EndoHf reveal Panx1-EGFP and Panx1∆VS-EGFP exhibited Gly0, Gly1, and Gly2 glycosylation species, while Panx1∆LRR-EGFP and Panx1∆HCS-EGFP exhibited only Gly0 and Gly1 forms. Anti-pan-cadherin and anti-β-actin were used as a positive and negative controls for deglycosylation, respectively. This figure was modified from Epp 2019^46^. For uncropped images of all Western blots in this figure, please see Supplementary Fig. S5.

**Figure 5:**
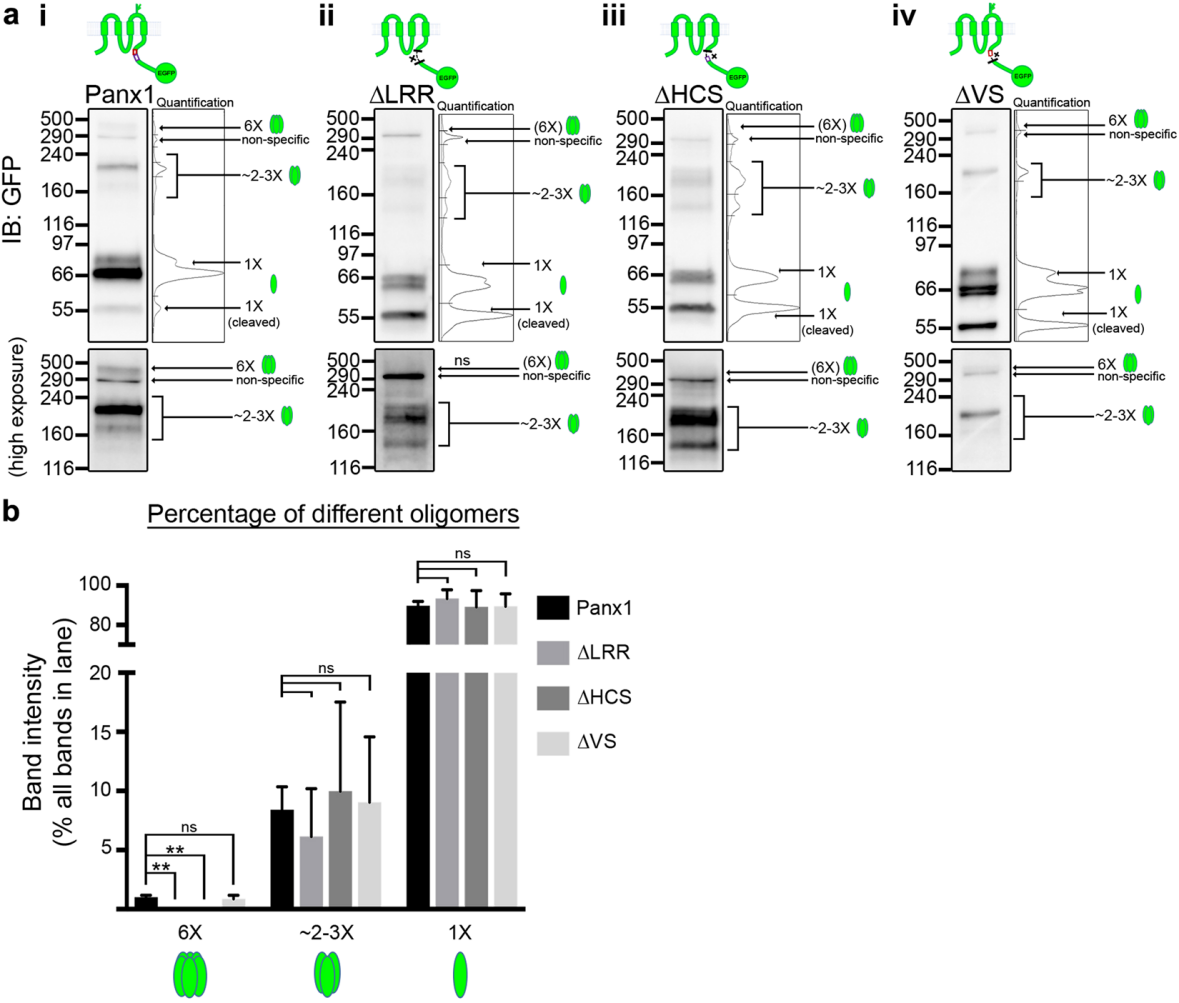
Surface hexamers are not detected with complete putative LRR motif or HCS sequence deletion. (**a**) Crosslinking assays reveal oligomerization profiles of LRR motif deletion mutants. Representative Western blots observing HEK293T cell lysates post-transfection with (*i*) Panx1-EGFP, (*ii*) Panx1∆LRR-EGFP, (*iii*) Panx1∆HCS-EGFP, or (*iv*) Panx1∆VS-EGFP, and after crosslinking with the cell-impermeable crosslinker, BS^3^, to observe surface-localized oligomers (6× or ~2–3×). Higher exposures of the regions (above 116 kDa) were included to better illustrate the presence or absence of high molecular weight bands. (**b**) Individual oligomeric bands were quantified and expressed as a percentage of the entire GFP signal in each lane. Non-specific bands were excluded from the analysis. Data are presented as mean ± SEM. One-way ANOVA with Dunnett’s multiple comparisons test*, N* = 3, α = 0.05, *F*(3,8) = 10.59, **P = 0.0072 (6×), ns, non-significant. All samples were derived from the same experiment and processed in parallel. This figure was modified from Epp 2019^46^. For uncropped images of all Western blots in this figure, please see Supplementary Fig. S6.

In light of the discovery of the putative LRR motif and the additional LRR HCS, together with previously reported shared ancestry and sequence conservation between Panx1 and LRRC8A, an LRR-containing protein^21^, we investigated the hypothesis that these C-terminal putative LRR motifs play a role in Panx1 cell surface localization. We started with the putative LRR motif at S328-K348 identified by ScanProsite (containing an HCS and an identified VS). We generated a full S328-K348 LRR motif deletion mutant (Panx1∆LRR-EGFP) as well as 2 partial LRR deletion mutants targeting separately the HCS at S328-L341 (Panx1∆HCS-EGFP) and the VS at K342-K348 (Panx1∆VS-EGFP; Fig. 4a). We then examined the localization and glycosylation states of these mutants. Panx1∆LRR-EGFP and Panx1∆HCS-EGFP exhibited drastically decreased surface localization, as determined by cell surface biotinylation (Fig. 4b) and confocal imaging (Fig. 4c). Additionally, only Gly0 and Gly1 forms were detected for Panx1∆LRR-EGFP and Panx1∆HCS-EGFP consistent with restricted cell surface expression (Fig. 4d). Conversely, Panx1∆VS-EGFP exhibited a slight yet significant decrease in subcellular distribution and had glycosylation profiles similar to full length Panx1-EGFP. Panx1∆VS-EGFP formed oligomers consistent with those of Panx1-EGFP (Fig. 5). Unexpectedly, we detected intermediate bands for Panx1∆LRR-EGFP and Panx1∆HCS-EGFP despite their drastic reduction in cell surface localization (and therefore reduced exposure to crosslinker). These intermediates were broader than those detected in Panx1-EGFP and Panx1∆VS-EGFP samples and were also faintly detectable in samples that had not been exposed to any crosslinker (data not shown).

Together, these data identify a putative LRR motif in the Panx1CT and reveal its critical role in Panx1 cell surface localization.

### Panx1-EGFP regulation of HEK293T cell proliferation requires the LRR motif

**Figure 6:**
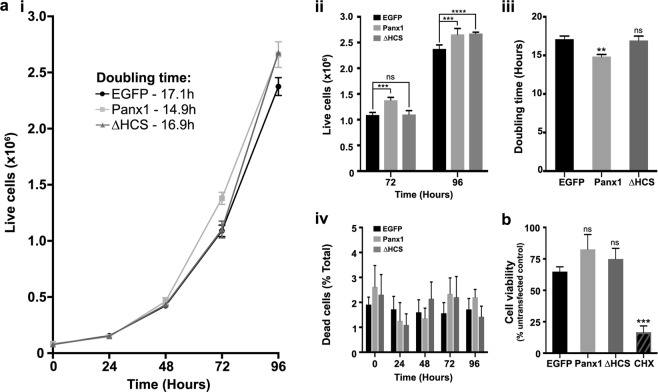
Panx1-EGFP overexpression increases HEK293T cell proliferation, but not when it lacks the HCS sequence (Panx1∆HCS-EGFP). (**a**) Trypan blue proliferation assays. (*i*) HEK293T cells overexpressing either Panx1-EGFP, Panx1∆HCS-EGFP, or EGFP control were counted at 5 time points with the first time point, T0, taking place at 24 h post-transfection (6 h after re-plating). Cell counts were used to plot growth curves. (*ii*) Histogram with cell counts from T0, T72, and T96. Data are presented as mean ± SEM. Two-way ANOVA with Dunnett’s multiple comparisons test, *N* = 4, α = 0.05, *F*(2,45) = 8.459, ***P = 0.0002, ****P < 0.0001, ns, non-significant. (*iii*) Doubling time comparisons of each condition, calculated using data from time points in the exponential growth phase. (*iv*) Dead cell counts at each time point, expressed as a percent of total cells. There were no significant differences in dead cell populations. Data are presented as mean ± SEM. One-way ANOVA with Dunnett’s multiple comparisons test, *N* = 4, α = 0.05, *F*(2,9) = 8.681, **P = 0.0079, ns, non-significant. (**b**) MTT assays performed at T72 on HEK293T cells overexpressing either EGFP, Panx1-EGFP, Panx1∆HCS-EGFP, or untransfected cells treated with a toxic dose (200 μg/mL) of cycloheximide (CHX) confirm that differences in cell numbers were not due to alterations in cell viability. Data are presented as the mean percentage of untransfected control ± SEM. One-way ANOVA with Dunnett’s multiple comparisons test, *N* = 6, α = 0.05, *F*(3,20) = 17.35, ****P < 0.0001, ns, non-significant. This figure was modified from Epp 2019^46^.

Our previous work suggested that Panx1 promotes N2a cell proliferation^22^ through as yet unknown mechanisms. Here we investigated the impact of expression of Panx1-EGFP, and in parallel expression of the Panx1∆HCS-EGFP deletion mutant exhibiting reduced cell surface localization, on the growth and proliferation of HEK293T cells (Fig. 6). Analysis of growth curves (Fig. 6ai,ii) revealed significant differences between Panx1-EGFP- and Panx1∆HCS-EGFP-expressing HEK293T cells. At 72 h, the abundance of Panx1-EGFP-expressing cells was significantly larger than that of EGFP-expressing control cells, whereas the abundance of Panx1∆HCS-EGFP-expressing cells was similar to control cell levels (Fig. 6ai,ii). By 96 h, however, both Panx1 wildtype and deletion mutant transfected cells exhibited similar cell counts, with both significantly more abundant than control cells (Fig. 6ai,ii). In terms of rate of cell division, Panx1-EGFP-expressing cells exhibited a significantly shorter doubling time than EGFP-expressing controls, while Panx1∆HCS-EGFP-expressing cells were not significantly different from controls (Fig. 6aiii). There were no differences in cell death or viability, as observed by the Trypan blue live/dead cell counts (Fig. 6aiv) and MTT assay (Fig. 6b). Overall, these data suggest the putative LRR motif is required for the effects of Panx1 on cell proliferation.

## Discussion

Discovery of a consensus sequence for a putative LRR motif within the proximal Panx1CT provides new mechanistic insight into the molecular determinants of Panx1 trafficking and localization. As we anticipated, the results of our cell surface expression and glycosylation state analyses were consistent, in that mature glycosylation was observed in Panx1 deletion mutants present primarily at the cell surface, while immature glycosylation was observed in Panx1 deletion mutants exhibiting markedly reduced cell surface expression. One possible interpretation of this finding is that deletion of the LRR or HCS (exhibiting markedly reduced cell surface expression) prevents trafficking to the *trans*-Golgi network (TGN), the site of complex glycosylation^23^, thereby preventing full glycosylation. In this scenario, glycosylation is not an active determinant of localization, but rather a passive bystander. On the other hand, it has previously been proposed that glycosylation actively directs localization^2–4^. This is, in part, based on the correlation between cell surface expression and full glycosylation amongst pannexin family members, in addition to markedly decreased cell-surface expression of glycosylation-deficient mutants. However, another possible mechanism to be investigated is that instead of driving ‘initial’ trafficking to the cell surface, complex glycosylation of Panx1 could impact on retention at the cell surface. Notably, our glycosylation state analysis also revealed that all deglycosylation enzyme-treated samples exhibited immunoreactive bands presenting ‘below’ the lowest ‘Gly0’ band of the untreated sample (actin negative control signals were unaffected). This finding suggests that the previously designated Gly0 species is glycosylated, potentially adding further complexity to our understanding of Panx1 glycosylation.

It should also be noted that we cannot rule out the possibility that the trafficking defects observed with expression of the putative LRR motif deletion mutants are secondary to impairment in subunit folding or oligomerization. Whether the impact of putative LRR motif deletion manifests through these indirect mechanisms, or rather through a more direct mechanism, such as a protein-protein interaction, will be the focus of future work. Importantly, we anticipate that global and targeted amino acid substitutions will help to reveal critical residues within this putative LRR motif and begin to shed light on the underlying mechanisms. Despite these un-answered questions, in stark contrast with the distal C-terminus this work nevertheless identifies the putative LRR motif as a key element of cell surface expression.

This new information on the role of the putative C-terminal LRR motif on Panx1 subcellular distribution has important implications for understanding the functional diversity of all 3 pannexin family members (Panx1, Panx2, and Panx3). With respect to the C-termini, the relatively weak homology between the 3 pannexins, compared with the homologies between other domains, suggests that the C-termini likely impart some unique aspects to their respective biological roles and regulation (localization, function, interactions, etc.). Panx1 and Panx3 exhibit relatively similar C-terminal (i.e. length, sequence homology) and both proteins traffic to the cell surface; whereas, Panx2 has a much larger C-terminus with low relative sequence homology, and localizes to intracellular compartments^4,24^. The Panx1 and Panx3 C-termini are also higher in leucine content than Panx2 (~15–17% compared to 8.8%, respectively). Examination of each sequence revealed multiple regions in the C-termini matching the LRR HCS consensus sequence (Fig. 3; Supplementary Fig. S2). It is reasonable to speculate that pannexin intermixing^4,25^ could involve interactions between putative larger LRR domains formed by the putative LRR motifs^13,26–29^.

To start to investigate the possibility of a larger LRR domain within Panx1, we examined the relationship between reported secondary structures commonly associated with LRR motifs/domains and the identified putative LRR and HCS motifs. Since an HCS usually forms a β-sheet and a VS often forms an α-helix^13,17,20^, we examined whether previously reported^30^ *in silico* identified putative secondary structures (partially confirmed using circular dichroism) in the Panx1CT directly aligned with HCS1-HCS5 (Fig. 3; Table 1). Notably, a previously identified β-strand^30^ aligned with HCS5 (Table 1), though it was not followed by an α-helix, as might be anticipated. The multiple HCS consensus sequences, the general promiscuity of LRR domain secondary structure^13^, and the previously identified mixture of α-helices and β-strands^30^ suggest the presence of a putative LRR domain within the Panx1CT.

**Table 1:** HCS consensus sequences in the Panx1CT. A list of amino acid sequences in the Panx1CT that match the broad LRR consensus rules: LxxLxLxxNxL or LxxLxLxxCxxL, where L is usually Leu, Iso, Val, or Phe (but can also be any hydrophobic amino acid); N is Cys, Ser, Thr, or Asn; C is Cys, Ser, or Asn; and x is any residue. Additionally, the first and last L in the sequence can be replaced by relatively hydrophilic residues. The HCS of the putative LRR motif examined in this study (HC2) is highlighted by a thick border outline.

Designated HCS number	Mode of discovery	Amino acids	Consensus sequence	Overlapping sites of interest
HCS1	Visual inspection	L305-L315	**L**kv**Y**e**I**lp**T**f**D**	Endocytic recognition sequence (Y308-L311)^47^ Phosphorylation site (Y308)^48^
HCS2	ScanProsite	L329-E340	**L**yn**L**f**L**ee**N**i**S** or **L**yn**L**f**L**ee**N**is**E**	Endocytic recognition sequence (Y330-F333)^47^
HCS3	Visual inspection	Y344-S355	**Y**kc**L**k**V**le**N**i**K** or **Y**kc**L**k**V**le**N**ik**S**	Endocytic recognition sequence (Y344-L347)^47^ Channel gating, S-Nitrosylation site (C346)^33^^,49^
HCS4	Visual inspection	I360-M371	**I**dp**M**l**L**lt**N**l**G** or **I**dp**M**l**L**lt**N**lg**M**	Putative membrane-interacting domain (I360-G370)^30^
HCS5	Visual inspection	I376-T387	**I**id**G**k**I**pt**S**l**Q** or **I**id**G**k**I**pt**S**lq**T**	Caspase-cleavage site (D375-D378)^34,35^ β-strand (K373-D380)^30^

In fact, LRRC8A, a volume-regulated anion channel containing LRR domains within its C-terminus, shares sequence homology with Panx1^21,28,29^. While the homology analysis between LRRC8A and Panx1 focused on the N-terminus to the fourth transmembrane domain^21^, our findings suggest that the degree of sequence and/or motif homology between C-termini could also be of potential interest. Notably, the LRRC8A hexamer is formed by a trimer of dimers^29^, reminiscent of the relatively abundant cell surface expressed ‘intermediate’ Panx1 oligomer(s) (Figs 2ai, 5ai) corresponding to either a dimer or trimer, previously detected in whole cell lysates^3,10^. A reasonable interpretation of our novel cell surface crosslinking data, is that Panx1 forms intermediate oligomers (dimers or trimers) that are stable at the cell surface.

As the Panx1CT regulates Panx1 activity (reviewed in Boyce *et al*.^1^, Chiu *et al*.^31^, Dahl 2018^32^), our discovery of a putative LRR motif in the Panx1CT could have functional implications. Several studies suggest that the C-terminus interacts with the Panx1 channel pore in an auto-inhibitory manner^33,34^. One group proposed a model in which a region in the Panx1CT, I360-G370, interacts with membranes, and that this interaction facilitates Panx1CT insertion into the Panx1 pore^30^. Interestingly, HCS4 almost directly overlaps with the suggested membrane-interacting region (Table 1). Further, HCS3 contains a cysteine group that was implicated in channel gating^33^, and HCS5 overlaps with the Panx1CT caspase-cleavage site which activates Panx1^34,35^. The potential impact of these putative HCS/LRR motifs on channel function warrants further study.

Here we observed a positive effect of Panx1 on cell growth in HEK293T cells consistent with our previous findings in N2a cells and neural precursor cells *in vitro*^22^ and *in vivo*^36^ and work from others on melanoma tumour growth^37^. It is important to note that in other systems (e.g. glioma^38^), Panx1 was instead found to suppress tumour growth. While our current understanding of the mechanisms underlying Panx1 regulation of cell growth and proliferation are limited, two reasonable theories are that Panx1 regulates cell proliferation via modulating cytoskeletal dynamics underlying cell growth and division via interactions with cytoskeleton-regulating proteins^5,39,40^, and/or purinergic signaling via mediating release of ATP^22,41–43^. It is important to note that these putative mechanisms are not mutually exclusive. Furthermore, more nuanced understanding of the role(s) of Panx1 in regulating cytoskeletal dynamics and purinergic signalling in various systems/contexts could help account for the differing observations. Moreover, regulation of cytoskeletal rearrangement might only partially depend on subcellular localization (for example deletion of the putative LRR motif could disrupt interactions with specific cytoplasmic proteins while leaving other interactions unperturbed). While more work is now needed to precisely determine the mechanistic underpinnings, our results support a previously identified role for Panx1 in regulating cell proliferation and implicate the newly discovered putative LRR motif.

Overall, the discovery of the putative LRR motif within the Panx1CT broadens our understanding of the molecular determinants of Panx1 trafficking and localization. This work also raises important new questions with respect to the regulation and functional implications of Panx1 oligomerization state at the cell surface.

## Methods

### Plasmids

The mouse Panx1-EGFP plasmid^2^ used in this study was a generous gift from Drs. Dale Laird and Silvia Penuela. Deletion mutants were generated using multiple rounds of PCR/overlap PCR followed by restriction digest and ligation into a vector backbone as previously described^7^. Briefly, overlap-extension PCR fragments were obtained from Panx1-EGFP using primers specific to regions up- and down-stream from the Panx1 gene and primers specific to regions on either side of the deletion site. The fragments were gel purified using the QIAquick Gel Extraction Kit (28704, Qiagen Sciences) and overlap-extension PCR was performed using the obtained fragments and primers for regions peripheral to the gene. The resulting PCR product was cloned into pEGFP-N1 vector. The last amino acid in the Panx1∆299-EGFP mutant is F298. The final amino acid in the Panx1∆379-EGFP deletion mutant is D378. All constructs were confirmed by sequencing (Eurofins MWG Operon LCC).

### Cell culture and transfection

Human Embryonic Kidney (HEK)-293T cells (CRL-3216™, ATCC) were cultured in Dulbecco’s modified Eagle’s medium (DMEM, 11965118) supplemented with 10% FBS (12483020), and 100 U/mL penicillin and 100 μg/mL streptomycin (15140122, all from Gibco/Life Technologies). Cells were transfected with 7.5 mM linear polyethylenamine (PEI) (23966, Polysciences) at a 10:1 PEI (μL):DNA (μg) ratio.

### Antibodies

Primary antibodies: anti-β-actin (1:16,000; A5441, Sigma), anti-EMMPRIN (1:1000; AF972-SP, R&D Systems), anti-GAPDH (1:6000; NB300-221, Novus Biologicals), anti-GFP mouse monoclonal (1:4000; 66002-1-lg, Proteintech), anti-GFP rabbit polyclonal (1:32,000; A6455, Molecular Probes), and anti-pan-cadherin (1:1000; 4068, Cell Signaling Technology). Secondary antibodies: horseradish peroxidase (HRP)-conjugated AffiniPure anti-rabbit immunoglobulin G (IgG, 711-035-152), HRP-conjugated AffiniPure anti-mouse IgG (715-035-150), and HRP-conjugated AffiniPure anti-goat IgG (705-035-003). All secondary antibodies were used at 1:8000 and obtained from Jackson ImmunoResearch.

### Protein extractions and Western blot

Proteins were extracted and analyzed by Western blot as previously described^22,39,44^. Unless otherwise specified, cells were harvested 48-h post-transfection by scraping in PBS 1% IGEPAL (10 mM PBS [150 mM NaCl, 9.1 mM Na_2_HPO_4_, 1.7 mM NaH_2_PO_4_], 1% IGEPAL CA-630) supplemented with protease inhibitor cocktail at 1 μL/10^6^ cells (stock: 0.104 mM 4-(2-aminoethyl)benzenesulfonyl fluoride hydrochloride, 0.08 mM aprotinin, 4 mM bestatin hydrochloride, 1.4 mM N-(trans-epoxysuccinyl)-L-leucine 4-guanidinobutylamide, 2 mM leupeptin hemisulfate salt, 1.5 mM pepstatin-A; P8340, Sigma), 0.2 mM PMSF, 10 μM sodium orthovanadate, and 1 mM EDTA and incubated on ice for 30 min. Homogenates were centrifuged 20 min at 12,000 rpm, and supernatants were collected. Samples were heated 5 min at 95 °C in the presence of 1X sample buffer and reducing agents (10% β-mercaptoethanol (BME) and 100 mM dithiothreitol (DTT)) prior to loading on an SDS-PAGE gel. Gels were 4–20% gradient gels (4561094, BioRad) for crosslinking assays, or 10% Laemmli gels for all other assays. Protein was transferred to a 0.2 μm pore-size polyvinyldene fluoride (PVDF; 1620177, BioRad) membrane. Membrane incubations were performed in 5% skim milk powder in PBS-T (10 mM Na_2_HPO_4_, 1.25 mM NaH_2_PO_4_, 2.7 mM KCl, 137 mM NaCl, 0.1% Tween-20). Blots were quantified by densitometry using ImageJ (http://imagej.nih.gov/ij/)^45^.

### Deglycosylation assays

Deglycosylation assays were performed using either PNGase F (P0704S, NEB) or EndoH_f_ (P0702S, NEB) according to the manufacturer’s protocol. Mouse anti-GFP (66002-1-lg, Proteintech) was used to detect proteins of interest.

### Cell surface biotinylation

Cells were washed twice with ice cold biotinylation buffer (137 mM NaCl, 2.7 mM KCl, 1.8 mM KH_2_PO_4_, 10 mM Na_2_HPO_4_, 0.5 mM MgCl_2_, 1 mM CaCl_2_; pH 7.4) then incubated in either 0.25 mg/mL EZ-Link^TM^ Sulfo-NHS-SS-Biotin (21331, Thermo-Fisher Scientific) in biotinylation buffer or buffer alone (negative control) for 30 min at 4 °C with gentle rocking. The reaction was quenched by adding 1 M glycine in biotinylation buffer to an end concentration of 100 mM glycine, then washing the cells twice (quick) and once for 15 min at 4 °C with gentle rocking in 100 mM glycine in biotinylation buffer. The solution was removed and lysates were prepared as described above, using TBS 1% IGEPAL (50 mM Tris, 150 mM NaCl, 1% IGEPAL CA-630; pH 7.5) and the aforementioned protease inhibitors as lysis buffer. As a preclearing step, lysates were incubated with a 50% slurry of 50 μL of Pierce^TM^ iminobiotin agarose beads (20221, Thermo-Fisher Scientific) for 1 h at 4 °C on with rotation. To isolate cell surface biotinylated proteins, 200–500 μg of total protein was incubated with 50% slurry of 50 μL of Pierce^TM^ NeutrAvidin™ agarose beads (29200, Thermo-Fisher Scientific) for 2 h at 4 °C with rotation. The beads were washed 4 times with TBS 1% IGEPAL, 4 times with high salt TBS 1% IGEPAL (50 mM Tris, 300 mM NaCl, 1% IGEPAL CA-630; pH 7.5), and twice with 50 mM Tris pH 7.5. Surface biotinylated proteins were eluted for 5 min at 95 °C on a heat block in 1X sample buffer and reducing agents (100 mM DTT and 10% BME). Eluates and inputs were analyzed by Western blot.

### Fixed cell imaging

Cells were plated onto poly-D-lysine (PDL; P6407, Sigma)-coated coverslips, 24 h post-transfection at a density of 7.5 × 10^4^ cells/cm^2^ and cultured for an additional 24 h period before collection. Directly before fixation cells were incubated with an AlexaFluor-647-conjugated cell surface marker wheat germ agglutinin (WGA; 5 μg/mL; Molecular Probes) in Hank’s Balanced Salt Solution (HBSS; 14170112, Gibco) for 5 min at 37 °C. Cells were quick-washed in phosphate buffered saline (PBS; 154 mM NaCl, 6.25 mM sodium phosphate monobasic, 18.75 mM sodium phosphate dibasic, pH 7.2), fixed with 4% paraformaldehyde (PFA) in PBS for 10 min, and washed thrice in PBS with the second wash containing Hoechst 33342 (1:300; H3570, Thermo Scientific) nuclear counterstain. Coverslips were mounted in VectaShield mounting medium (H-1000, Vector Laboratories, Inc.). Images were acquired with a Leica TCS SP8 confocal microscope using a 40× (1.3 NA) oil immersion objective (1024 × 1024, 2X optical zoom, pixel area 20 nm^2^) as 10 µm confocal z-stacks (z-size 0.5 µm) with the center of the z-stack corresponding to the optical section containing the largest central plane of most nuclei in the field of view (FOV), obtained using a contrast-based autofocus method within the Leica Application Suite (version 3.5.5., Leica Microsystems GmbH). This central optical section was used for all co-distribution (Pearson’s Correlation Coefficient) and colocalization rate (% colocalization area/foreground area) analyses performed within the Leica Application Suite software. Representative images were adjusted uniformly using Adobe Photoshop (CC 2018, 19.1.1). Imaging and analysis were performed by a researcher blind to experimental conditions.

### BS^3^ crosslinking assays

To observe mature protein (enriched at the cell surface), transfected cells were treated with 20 μg/mL cycloheximide (CHX; C7698, Sigma) for 8 h prior to crosslinking. Cells were washed with ice-cold crosslinking buffer (20 mM PBS [150 mM NaCl, 18.6 mM Na_2_HPO_4_, 1.4 mM NaH_2_PO_4_], 1 mM CaCl_2_, 0.5 mM MgCl_2_; pH 8), and incubated in either 3 mM bis(sulfosuccinimidyl)suberate (BS^3^; 21580, Thermo-Fisher Scientific) in crosslinking buffer or buffer alone for 30 min on ice. The reaction was quenched by adding 1 M Tris to an end concentration of 20 mM, then incubating in quenching solution (20 mM Tris, 20 mM PBS [150 mM NaCl, 13.7 mM Na_2_HPO_4_, 6.3 mM NaH_2_PO_4_], 1 mM CaCl_2_, 0.5 mM MgCl_2_; pH 7.2) for 15 min on ice. The solution was removed and lysates were prepared and analyzed by Western blot as described above.

### Trypan blue proliferation and MTT assays

Cells were transfected as described above and, at 18 h post-transfection, replated onto 35 mm dishes at 1 × 10^5^ cells/dish for Trypan blue proliferation assays or in 96-well plates at 1 × 10^4^ cells/well for MTT assays. Cells were counted every 24 h for 5 days (T0-96), with T0 occurring at 24 h post-transfection. Doubling time was calculated [DT = T * ln2/ln(N_T=1_/N_T=2_)] using time points within the linear logarithmic growth curve. MTT assays were performed at T72 using the Vybrant® MTT Cell Proliferation Assay Kit (V13154, Thermo-Fisher Scientific), according the manufacturer’s protocol.

### Statistical analysis

Statistical analysis was performed using Prism 7 (GraphPad Software). The tests and P values for each experiment are described in figure legends.

## Supplementary information

Supplementary Information

## Data Availability

The raw data or materials generated and/or analyzed during the current study are available from the corresponding author on reasonable request.
