# Supplementary material for: A novel motif in the proximal C-terminus of Pannexin 1 regulates cell surface localization
Source: Sci Rep. 2019 Jul 5;9:9721. doi: 10.1038/s41598-019-46144-5 (PMC6611761; doi:10.1038/s41598-019-46144-5)
Supplement: Supplementary file 1 — Supplementary Information [file 41598_2019_46144_MOESM1_ESM.pdf]

Supplementary Information

A novel motif in the proximal C-terminus of Pannexin 1 regulates cell surface localization

Anna L. Epp[1], Sarah N. Ebert[1], Juan C. Sanchez-Arias[1], Leigh E. Wicki-Stordeur[1], Andrew K.J. Boyce[1], Leigh Anne Swayne[1,*]

[1]Division of Medical Sciences and Island Medical Program, University of Victoria, Victoria, V8P 5C2, Canada

*Corresponding author at: Division of Medical Sciences, 3800 Finnerty Rd, Victoria, BC, Canada. Email address: lswayne@uvic.ca (L.A. Swayne); twitter: @dr_swayne

Legend to Supplementary Figures

Supplementary Figure S1. ***Expression levels of all Panx1 deletion mutants.*** Expression levels were determined from the input lanes of cell surface biotinylation Western blot analyses. Signals from anti-GFP (protein of interest) and anti-EMMPRIN (positive and loading control) were quantified, normalized, and expressed as percent of full length Panx1-EGFP. Data are presented as mean ± SEM. One-way ANOVA with Dunnett's multiple comparisons test, $N = 6$, $\alpha = 0.05$, ****$P<0.0001$.

Supplementary Figure S2. ***HCS consensus sequences in the other pannexin family member C-termini.*** Sequence alignment of (A) Panx2CT and (B) Panx3CT using sequences from human, mouse, and rat, with regions of each identified HCS highlighted in red. The alignment was generated in CLUSTAL O (1.2.3) using NCBI accession numbers: NP_443071.2 (human), NP_001002005.2 (mouse), and NP_955441.2 (rat) for Panx2, and NP_766042.1 (human), NP_4431931.1 (mouse), and NP_955430.1 (rat) for Panx3.

Supplementary Figure S3. ***Uncropped images of Western blots presented in Figure 1.*** Western blots from (a) cell surface biotinylation assays, (b) PNGase F deglycosylation assays, and (c)

Endo Hf deglycosylation assays. The boxed images are those that are shown in Figure 1b,d. Each blot was stripped prior to being reprobed with a different antibody (black arrows).

Supplementary Figure S4. ***Uncropped images of Western blots presented in Figure 2.*** The boxed images are those that are shown in Figure 2.

Supplementary Figure S5. ***Uncropped images of Western blots presented in Figure 4.*** Western blots from (a,b) cell surface biotinylation assays, (c) PNGase F deglycosylation assays, and (d) Endo Hf deglycosylation assays. The boxed images are those that are shown in Figure 4b,d. Each blot was stripped prior to being reprobed with a different antibody (black arrows).

Supplementary Figure S6. ***Uncropped images of Western blots presented in Figure 5.*** The boxed images are those that are shown in Figure 5.

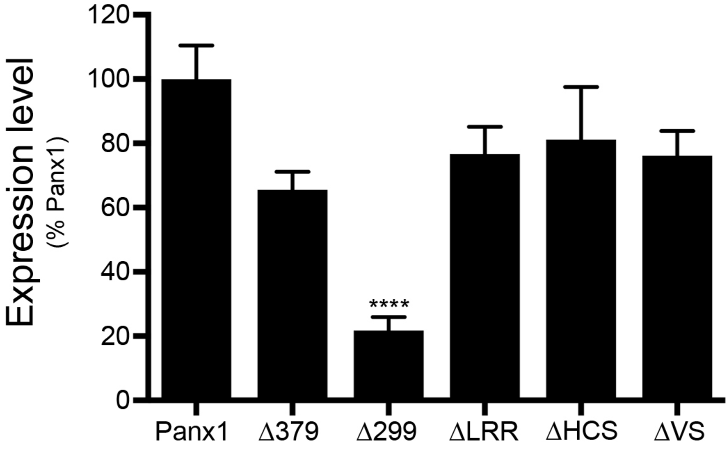
Expression level
(% Panx1)
0
20
40
60
80
100
120
****
Panx1
Δ379
Δ299
ΔLRR
ΔHCS
ΔVS

Supplementary Figure S2. Epp *et al.*, 2019

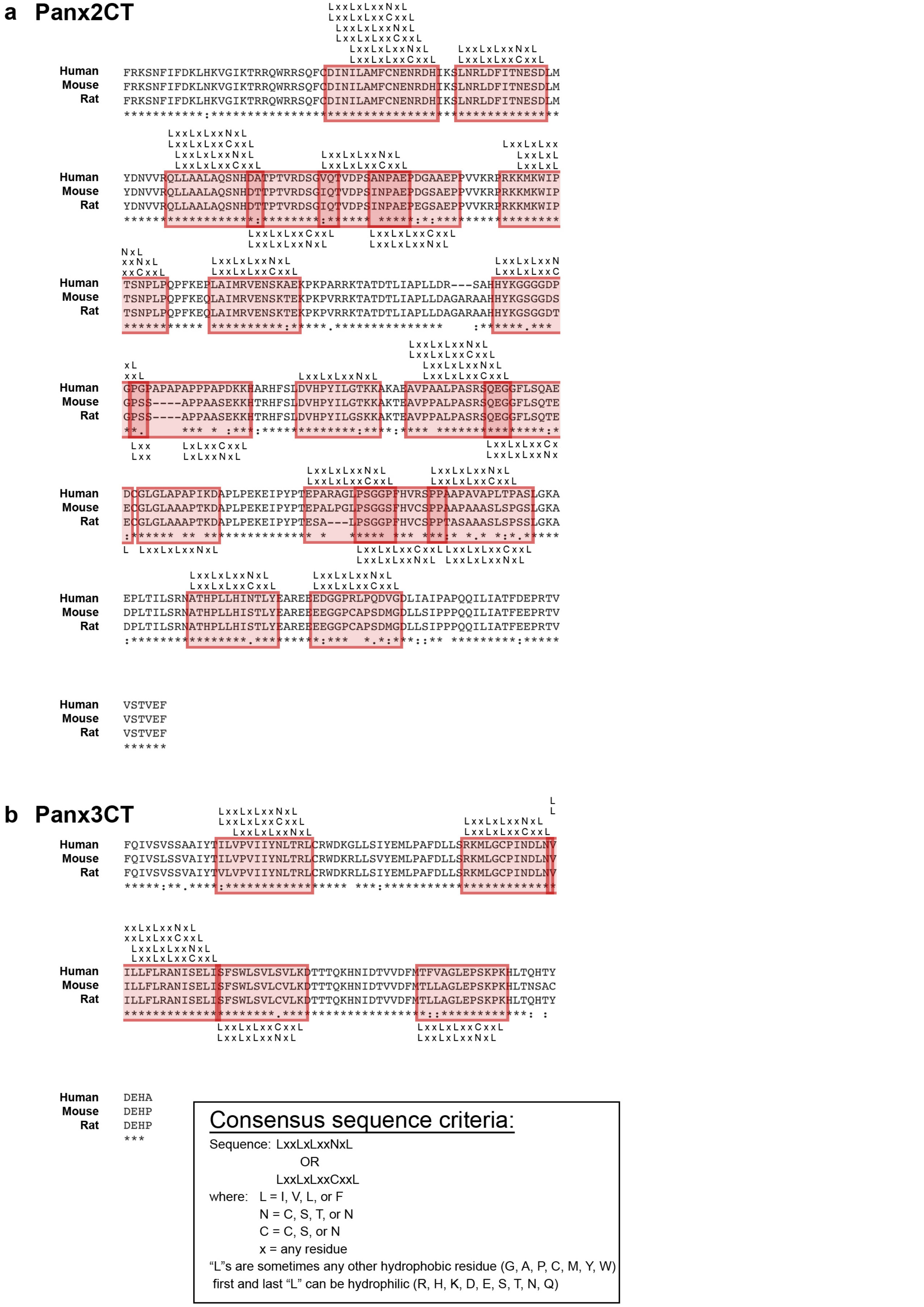

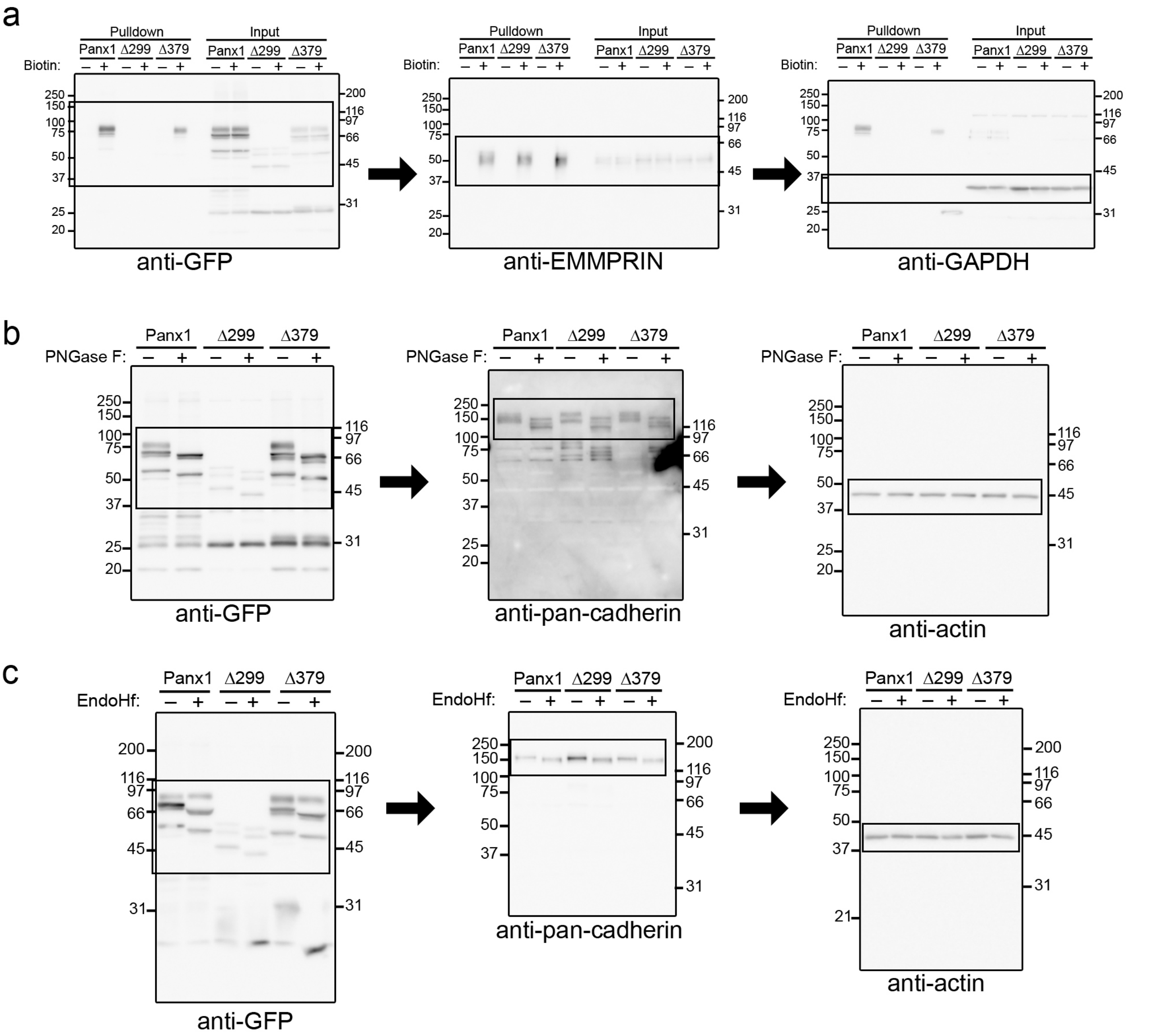
a
Pulldown
Input
Panx1 Δ299 Δ379
Biotin:
anti-GFP
anti-EMMPRIN
anti-GAPDH
b
PNGase F:
anti-GFP
anti-pan-cadherin
anti-actin
c
EndoHf:
anti-GFP
anti-pan-cadherin
anti-actin

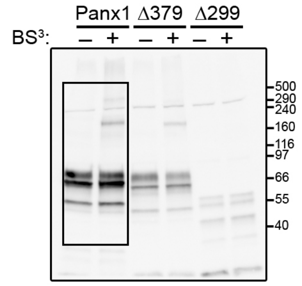

anti-GFP
(shorter exposure)

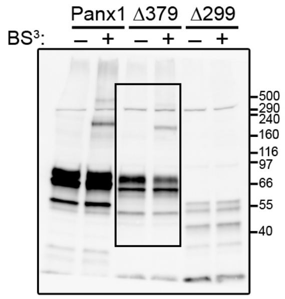

anti-GFP
(medium exposure)

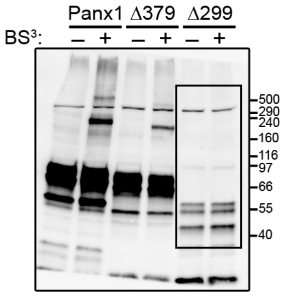

anti-GFP
(longer exposure)

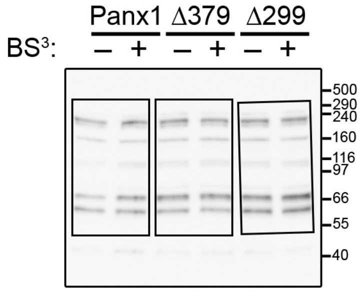

anti-Crmp2

Supplementary Figure S5. Epp *et al.*, 2019

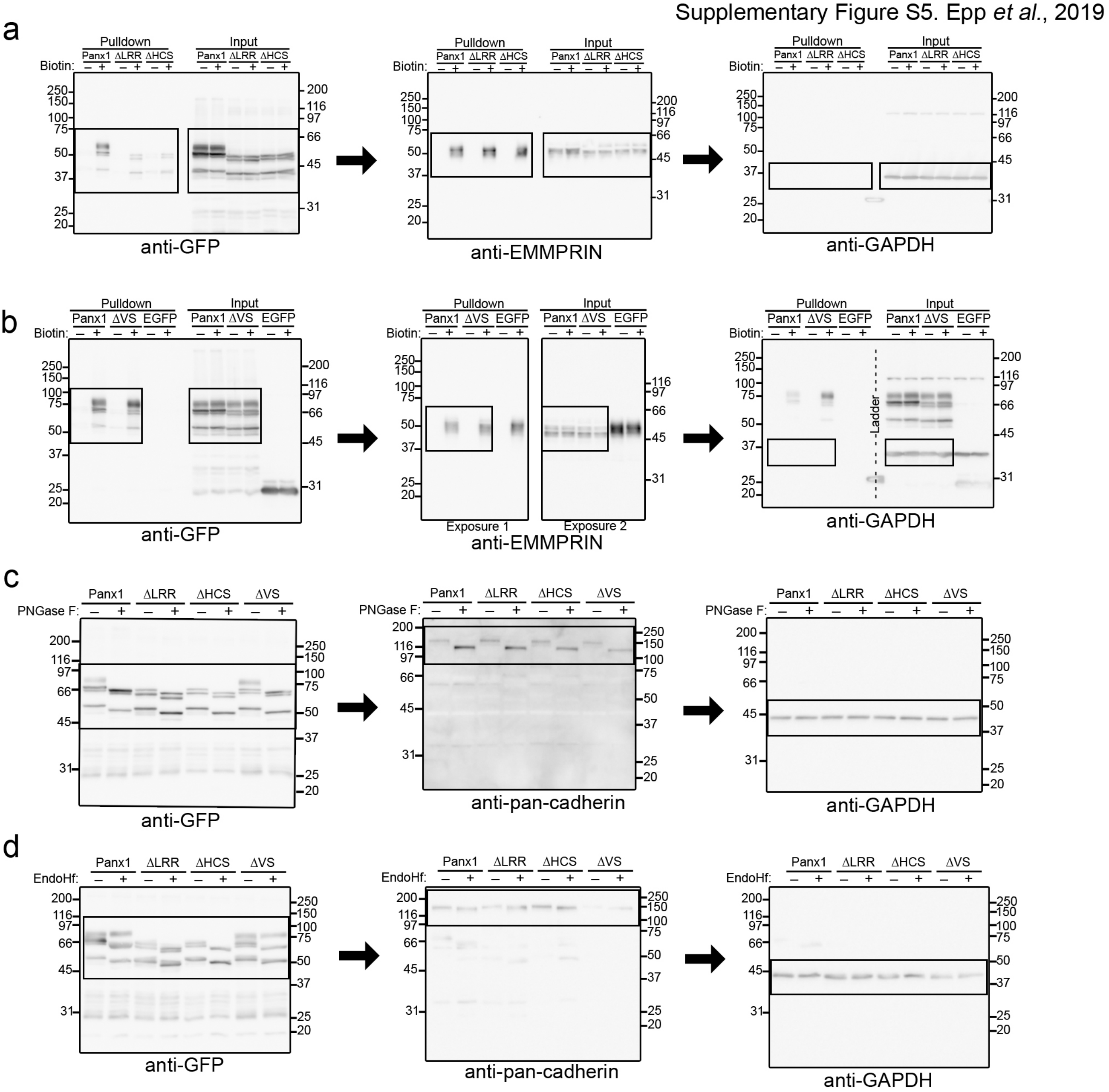

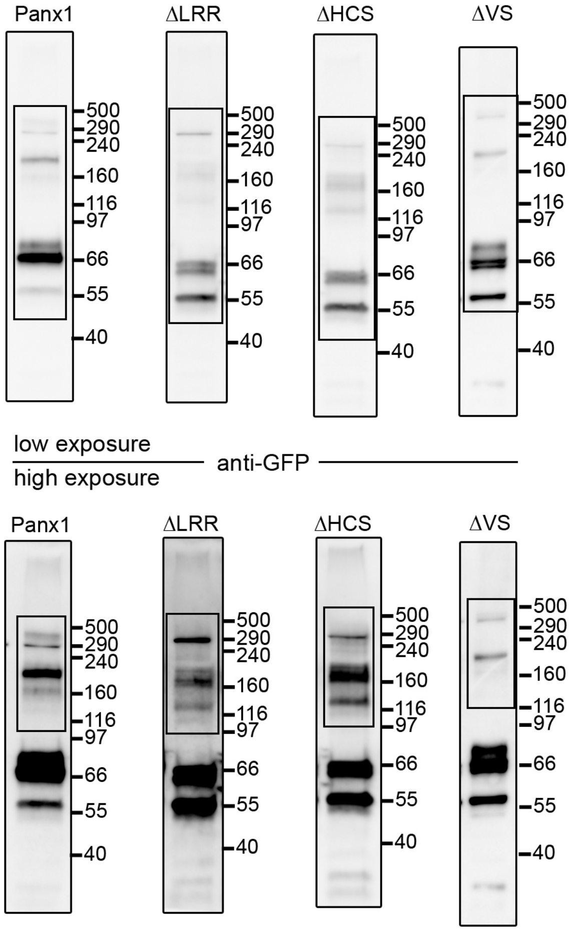
Panx1
ΔLRR
ΔHCS
ΔVS
500
290
240
160
116
97
66
55
40
low exposure
anti-GFP
high exposure
Panx1
ΔLRR
ΔHCS
ΔVS
500
290
240
160
116
97
66
55
40
